# 아로마테라피가 산전 및 산후 피로에 미치는 영향: 체계적 문헌고찰 및 메타분석

**DOI:** 10.4069/kjwhn.2022.05.15

**Published:** 2022-06-16

**Authors:** Ji-Ah Song, Hyejin Yang

**Affiliations:** College of Nursing, Konyang University, Daejeon, Korea; 건양대학교 간호대학

**Keywords:** Aromatherapy, Fatigue, Postnatal care, Prenatal care, Systematic review, 아로마테라피, 피로, 산후, 산전, 체계적 문헌고찰

## Abstract

**Purpose:**

The purpose of this systematic review was to examine aromatherapy interventions for prenatal and postnatal women, and to determine the effectiveness of these interventions on fatigue.

**Methods:**

Six national and international databases were reviewed to retrieve and collect studies published up to September 7, 2021, describing randomized controlled trials and controlled clinical trials of aromatherapy interventions for prenatal and postnatal women’s fatigue. Of the 323 articles initially identified, 64 duplicates were excluded and 259 were screened. After further excluding 216 articles not related to PICO framework, 10 were selected for review. Two reviewers independently selected studies and conducted data extraction and quality appraisal using Cochran’s Risk of Bias and Risk of Bias Assessment Tool for Non-randomized Studies.

**Results:**

The quality of the 10 selected studies was overall satisfactory. A meta-analysis of three studies showed that aromatherapy with lavender oil? produced a 0.75-point reduction in postnatal mothers’ fatigue when compared to control groups. Sleep quality was also analyzed as a secondary outcome of fatigue. A meta-analysis of four studies using lavender and/or orange peel oil found that aromatherapy produced a 0.98-point improvement in postnatal mothers’ quality of sleep. Although a meta-analysis could not be conducted to synthesize the findings for fatigue in pregnant women, inhalation and massage therapy using lavender oil showed positive effects on prenatal fatigue and sleep quality.

**Conclusion:**

Aromatherapy using lavender oil and orange peel oil is effective in improving prenatal and postnatal fatigue and sleep quality.

## Introduction

산과적 피로는 산모의 정서적 안정과 미래의 행위에 영향을 미치는 산전, 산후 증상이다[1]. 피로는 몸의 에너지 불균형과 에너지 수급이 부조화의 결과로 발생하며, 피로와 에너지 부족은 임신과 산후 기간에 자주 보고된다[2]. 이란에서는 19–29주 초임부의 54.8%에서 중간 정도의 피로를 호소하였으며, 7.1%에서는 심한 피로를 호소하였다[3]. 대만의 또 다른 연구에서는 임신 25–29주 임부의 41.1%, 29–34주 39.4%, 34–38주 45.5%가 피로 증상을 경험했다고 보고하였다[4]. 또한 36주 이상의 임부 중 75%가 피로를 경험하였으며, 25%의 임부는 심한 피로를 호소하였음을 보고하였다[5]. 터키의 연구에서는 10–14주 임부의 93.3%가 피로를 경험했으며, 22–24주 임부는 96.7%, 34주 이후 임부는 모두 피로를 경험하였다[6]. 이러한 산전 피로는 자궁기능 부전과 자궁경관 개대 억제, 그리고 분만 1기 활동기의 장애 원인으로 알려져 있으며[7], 외상 후 스트레스 장애[8], 이후 임신에서의 산전‧산후 우울증과 제왕절개 요구[9]와 같은 부정적인 산과적 경험을 초래할 수 있다.

산모들은 긴 시간 분만으로 인해 피로를 느끼고, 모유 수유와 영아 돌봄으로 인해 수면시간이 부족하여 피로는 더욱 증가한다[10,11]. 또한 수면 관련 문제와 통증, 치질, 변비, 유방 문제 등과 같은 육체적 탈진을 경험함으로써 피로는 주요 관심사로 대두되었다[11,12]. 피로는 신체와 정신적 활동의 소모와 능력 감소에 대한 주관적인 보고로 정의되며[13], 초기 산욕기 산모의 80%에서 보고되고 있다[14]. Khayamim 등[15]의 연구에서는 59.5%의 산모가 중간 수준의 피로를 경험했고, 35.3%에서는 심한 피로감을 겪었다고 보고하였다. 가장 일반적으로 겪는 산욕기 산모들의 문제는 피로와 수면 장애, 배뇨 장애로 보고되고 있는데[16], 수면 문제와 모유 수유, 불안감은 피로를 증가시키며[11,17], 산후우울증과 유의한 관련이 있어 산후우울증을 예측하는 지표이다[18].

아로마테라피(aromatherapy)는 여러 증상을 관리하기 위해 전 세계적으로 널리 사용하는 보완대체요법으로[19,20] 식물에서 추출한 에센셜 오일(essential oil)을 사용하여 후각이나 피부 흡수를 통해 생리적 또는 약리적 효과를 내는 과정으로 정의한다[21]. 에센셜 오일은 항균, 항염증, 진통, 항독성, 면역 항진 및 암, 호흡기 질환, 편두통, 고혈압, 관절염, 근육 관련 통증 등의 증상을 관리하고 완화하기 위해 사용해 왔다[21]. 또한 암 환자와 관절염 환자, 투석 환자와 같은 만성질환자의 피로를 감소시키고[22-24], 중년 여성, 학생들의 피로를 감소시키는 효과뿐 아니라[25,26] 수면의 질에도 긍정적인 효과가 있다고 보고되었다[27].

산모에게 적용한 아로마테라피의 선행연구를 살펴보면, 산욕기 산모에게 아로마 오일을 이용한 족욕과 손 마사지를 적용한 결과 피로 및 산후 우울증의 억제효과가 있었으며[28,29], 아로마 오일 흡입을 제공 받은 산모는 수면의 질이 향상되었다[30,31]. 또한 다양한 연구에서 아로마테라피가 임신과 산후조리 과정에서 삶의 질 뿐만 아니라 우울증, 불안감 등 다양한 증상을 치료하고 개선하는 데 이용된다는 사실이 밝혀졌다[30,32]. 이러한 연구들의 결과로 아로마테라피는 피로를 겪는 산전 및 산후 산모들에게 적합하다 생각되며, 이를 확인하고자 한다. 또한 아로마 오일의 사용 방법, 종류, 양, 사용기간 등에 있어서 연구마다 다양하고 차이가 있어 실제 임상에 적용하기에 제한이 있다. 따라서 본 연구의 목적은 아로마테라피가 산전 및 산후 피로에 미치는 영향을 근거에 기반하여 체계적으로 고찰하고, 이용 가능한 간호 중재로 결정하는 데 있다. 또한 본 연구는 산전 및 산후 피로 증상을 줄이고 삶의 질을 향상시키기 위한 노력을 기울이는 보건 전문가와 여성들에게 잠재적으로 유용하다 할 수 있다.

## Methods

Ethics statement: This study was exempted by the Institutional Review Board of Konyang University (KYU-NON2021-001) as it was analysis of existing literature.

### 연구 설계

본 연구는 아로마테라피가 산전 및 산후 산모의 피로에 미치는 효과를 검증한 실험연구들을 분석한 체계적 문헌고찰이다.

### 핵심 질문(PICO-SD)

#### 연구 대상(patients)

본 연구는 임신 및 출산 후 여성을 대상으로 하였다.

#### 중재(intervention)

본 연구에서는 방향성 실물의 추출물이나 에센셜 오일을 이용하여 피부 또는 호흡기를 통해 체내에 흡수시킨 아로마테라피를 적용한 연구들을 선정하였다.

#### 비교 중재(comparator)

본 연구의 비교중재로는 아로마테라피를 제공하지 않는 대조군과 아로마 오일이 아닌 플라시보 오일(placebo oil)을 적용한 비교군을 선정하여 분석하였다.

#### 중재 결과(outcome)

중재 결과는 아로마테라피가 산전 및 산후 피로에 미치는 효과를 확인하기 위한 것으로 일차 중재 결과로는 피로를 선정하였으며, 이차 중재 결과는 수면의 질을 선정하여 분석하였다.

#### 연구 설계(study design)

본 연구의 연구 설계는 무작위 대조군 실험연구(randomized controlled trial, RCT) 및 대조군 임상연구(nonrandomized controlled clinical trial, CCT)를 대상으로 하였다.

### 자료 검색, 수집 및 선별 절차

#### 자료 검색

자료 검색은 2021년 9월 7일 이전까지 검색 가능한 모든 문헌을 검색하였다. 검색에 활용된 국외 데이터베이스는 PubMed, CINAHL (Cumulative Index of Nursing and Allied Health Literature), CENTRAL (Cochrane Central Register of Controlled Trials), 국내 데이터베이스는 RISS (Research Information Service System), KISS (Korean Studies Information Service System), DBpia를 통하여 출판된 학술지 논문을 검색하였으며, Google Scholar을 이용하여 수기 검색을 진행하였다. 검색식은 MeSH (Medical Subject Headings) 용어와 text word를 불리언 연산자(Boolean operators)인 AND, OR 및 절단 검색을 적절히 사용하였으며, 데이터베이스 별 ‘clinical trials’, ‘text word’ 등과 같은 필터를 사용하여 민감도를 높였다. 먼저 중재 방법으로 ‘aroma*’, ‘aromatherapy [MeSH]’, ‘essential oil’의 용어를 사용한 문헌을 모두 추출하였고, 연구대상자는 ‘prenatal’, ‘postnatal’, ‘pregnancy [MeSH]’, ‘postpartum period [MeSH]’인 문헌을 검색하였다. 문헌의 민감도를 높이기 위해 비교 중재는 제한하지 않았으며, 중재 결과는 ‘fatigue [MeSH]’로 검색하였다. 결론적으로 검색식은 (aroma* OR aromatherapy [MeSH] OR essential oil) AND (prenatal OR postnatal OR pregnancy [MeSH] OR postpartum period [MeSH]) AND figure [MeSH]였다. 아로마테라피는 국내에서 향기 요법이라는 용어로도 사용되기 때문에 국내 데이터베이스에서도 같은 방법으로 아로마, 아로마테라피, 향기요법, 아로마 오일, 산전, 산후, 산모, 산욕기, 피로 등 개념어를 넣어 검색하였다. 논문은 PRISMA (Preferred Reporting Items for Systematic Reviews and Meta-Analyses) 2020 보고지침[33]에 따라 기술하였다.

#### 자료 수집과 선별

본 연구의 문헌 선정 기준은 다음과 같다. (1) 산전 및 산후 피로에 대한 아로마테라피 효과 연구, (2) RCT 및 CCT (3) 학술지에 출판된 논문, (4) 한국어와 영어로 출판된 연구를 포함하였다. 배제 기준은 다음과 같다. (1) 아로마 오일을 피부 또는 호흡기를 통해 체내에 흡수시키지 않은 연구, (2) 분만 중인 산부를 대상으로 한 연구, (3) 동물실험 또는 전 임상시험 연구, (4) 비(非) 비교 연구(non-comparative study), (5) 출판되지 않은 학위 논문, (6) 관찰연구, 종설 연구는 제외하였다.

**Figure 1.**
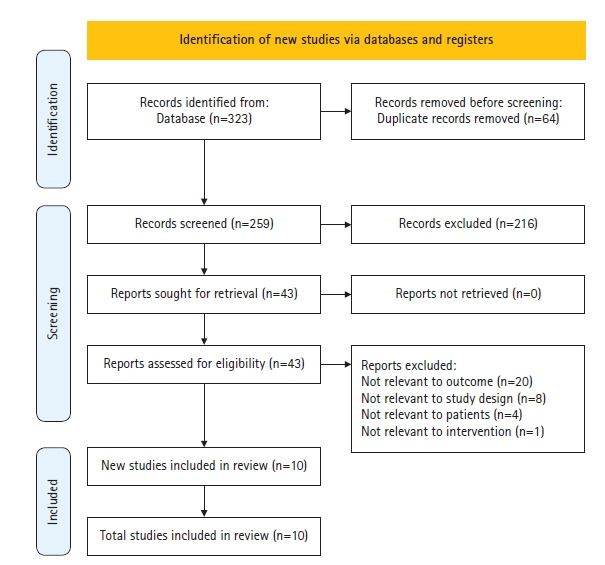
Flow chart of the study selection process.

본 연구는 검색된 자료를 핵심 질문과 선정 및 배제 기준을 바탕으로 연구자 2명이 독립적으로 검토하여 선별하였다. 의견 불일치 시에는 충분한 논의와 제 3의 연구자의 의견을 수렴하는 과정을 거쳐 문헌을 선정하였으며, 단계별 문헌 선택 과정을 기술하기 위해 PRISMA 2020 흐름도를 사용하였다[33]. 문헌 검색 결과 PubMed 189편, CINAHL 56편, Cochrane 48편, DBpia 6편, KISS 2편, RISS 10편이 검색되었으며, Google Scholar를 이용한 수기 검색 결과는 12편으로 총 323편이 검색되었다. 이 중 중복된 64편을 제외한 259편의 문헌을 대상으로 2명의 연구자가 제목과 초록을 중심으로 검토하였다. 그 결과 핵심 질문과 관계 없는 연구와 연구 설계가 선정기준에 부합하지 않은 연구 등 총 216편을 제외한 43편을 1차 선별하였다. 1차 선별된 43편의 문헌을 원문 중심으로 동일한 기준과 과정에 따라 검토한 결과 피로 효과가 아닌 연구 20편, 연구 설계가 부합하지 않은 연구 8편, 산전 및 산후 산모 대상이 아닌 연구 2편, 분만 중인 산모 연구 2편, 아로마테라피가 아닌 연구 1편을 제외한 총 10편을 2차 선별하였다. RCT 중 결과 변수가 평균과 표준편차로 제시되어 있는 연구 5편에 대해서 메타 분석을 하여 양적 분석을 시행하였다(Figure 1).

### 문헌의 질 평가

본 연구에서는 Cochrane의 무작위 배정 비교임상시험연구 질 평가 도구인 RoB (Risk of Bias)와 비무작위 연구의 질 평가 도구인 RoBANS (Risk of Bias Assessment tool of Non-randomized Study)를 사용하여 문헌의 비판적 검토를 시행하였다[33]. 평가 방법은 질 평가 항목에 대하여 그 위험성의 정도를 낮음, 불확실, 높음으로 평가하였으며, 최종 선별된 문헌을 대상으로 2명의 연구자가 독립적으로 시행하였고, 일치하지 않는 항목이 있는 경우 제3의 연구자의 의견을 듣고 충분한 논의를 거쳐 일치된 결과로 도출하였다.

### 자료 추출

체계적 문헌 고찰에 포함된 10편의 연구의 특성을 분석한 후 코드화하여 정리하였다. 추출표에는 1저자, 출판연도, 연구설계, 실험군 및 대조군에 대한 중재방법과 표본 수, 아로마 오일의 종류, 결과 변수, 그룹 간 차이, 저자의 결론으로 구성하였다.

### 자료 합성

선정된 연구에 대한 체계적 확인, 합성, 통계적 병합 및 결과 보고는 Cochrane 핸드북을 바탕으로 분석하였으며[33], 효과 크기는 RevMan software ver 5.4. (Cochrane Collaboration, London, UK)을 사용하여 분석하였다. 선정된 연구는 사용한 오일의 종류와 중재 방법 측면에서 이질성이 존재한다고 판단되어 변량효과모형(random-effects model)을 사용하여 분석하였다. 또한 결과 변수가 연속형 변수이므로 평균과 표준편차를 사용하였으며, 연구 도구는 다양하였으므로 표준화된 평균 차(standardized mean difference, SMD)를 사용하여 분석하였다. 각각의 결과 변수의 효과와 95% 신뢰구간(95% confidence interval, 95% CI)은 역 분산(inverse variance) 방법을 사용하여 분석하였다.

또한 연구의 이질성을 결정하기 위해 연구 간 신뢰구간과 효과 추정치에 공통적인 부분을 확인하는 숲 그림을 통해 시각화하였으며, 통계적 검정 방법으로 Higgins I²-statistic을 사용하여 연구의 이질성을 확인하였다. I²값이 25% 이하인 경우 이질성이 낮다, 50%에서 75%미만인 경우 이질성이 중간이다, 75% 이상인 경우 이질성이 높다고 판단하였으며, 중간 정도의 이질성이 있는 경우 그 원인을 탐색하기 위하여 소그룹 분석을 시행하였다. 본 연구에서 메타 분석에 포함된 연구는 총 5편으로 편으로 Funnel plot의 비대칭 여부에 대한 해석은 포함된 연구들이 적어도 10개 이상인 경우에만 적용될 수 있다는 근거에 따라 출판 비뚤림은 판단할 수 없었다[33].

## Results

### 체계적 문헌고찰 대상 논문의 일반적 특징

본 연구의 자료 선정 및 배제 기준에 따라 체계적 문헌고찰에 포함된 연구 10편[29-31,34-40]의 특성은 다음과 같다(Tables 1,2).

분석에 포함된 연구는 RCT 6편[30,31,34-37], CCT 4편[29,38-40]이었으며, 임신 여성을 대상으로 한 연구 2편[34,40], 산후 여성을 대상으로 한 연구 7편[29,30,35-39], 임신 및 산후 여성 모두를 대상으로 한 연구는 1편이었다[31]. 아로마테라피 중재 방법으로는 비강으로 흡수시키는 흡입 방법이 7편[30,34-37,39,40], 피부로 흡수시키는 마사지 방법 3편[29,31,38]이었으며, 그중 1편[35]은 아로마 차를 이용하여 흡입과 구강을 통해 체내로 흡수시키는 방법을 동시에 사용하였다. 사용한 아로마 오일은 라벤더(lavender) 오일을 사용한 연구가 9편으로[29-31,34,35,37-40] 가장 많았으며, 그 밖에 일랑일랑(ylang-ylang), 시트론(citron), 로즈우드(rosewood), 스위트 오렌지(sweet orange), 버가못(bergamot), 샌달우드(sandalwood), 오렌지(orange) 오일을 단독 또는 혼합하여 사용하였다[36,38]. 아로마 오일을 단독으로 사용한 연구는 7편[30,31,34,35,37,39,40]으로 단독 연구에서 사용된 오일은 라벤더였으며, 중재 기간을 살펴보면 1회부터 최대 8주까지 다양하게 나타났다. 피로를 측정하기 위한 도구는 다양하게 나타났는데, Postpartum Fatigue Scale [35], Multidimensional Assessment of Fatigue [31], Profile of Mood States [30], visual analogue scale [37,40], Fatigue Assessment Scale [39] 등의 도구가 사용되었다. 수면의 질을 측정하기 위한 도구로는 Pittsburgh Sleep Quality Index [30,31,36] 도구가 가장 많이 사용되었으며, 그 외 Postpartum Sleep Quality Scale [35] 등의 도구가 사용되었다.

### 문헌의 질 평가

#### RoB 질 평가 결과

**Figure 2.**
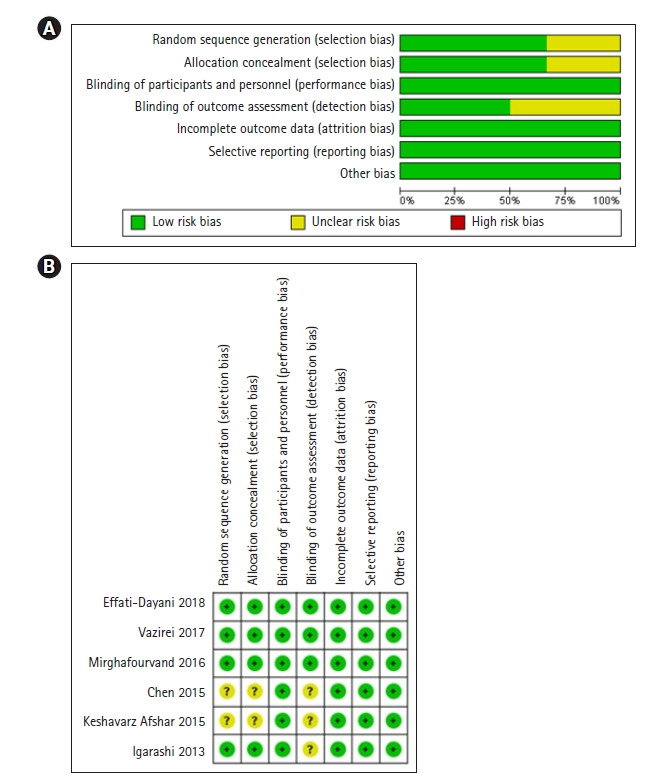
RIsk of bias for selected randomized controlled trials studies. (A) Risk of bias graph and (B) risk of bias summary.

RCT 6편의 문헌에 대한 질 평가는 다음과 같다(Figure 2). 참여자와 연구자에 대한 눈가림, 불충분한 자료, 선택적 결과 보고 항목에서 비뚤림은 나타나지 않았는데, 이는 포함된 연구가 사전 설정된 프로토콜에 따라 진행되었음을 시사했다. 무작위 배정 순서 생성, 배정 순서 은폐 항목에서 낮은 위험을 가진 연구는 4편(66.7%)이었으며[31,34,36,37], 결과 평가에 대한 눈가림 항목에서 낮은 위험을 가진 연구는 3편(50.0%)이었다[31,36,37]. 몇몇 연구에서는 이러한 비뚤림 위험을 언급하지 않았기 때문에 비뚤림 위험을 판단할 수 없었다. 오일의 독특한 향을 내는 아로마테라피의 특성상 참가자와 연구자, 결과 평가자의 눈가림을 하기 어려운 것은 사실이므로 단일 눈가림 연구가 대부분이었다. 그럼에도 불구하고 이중 맹검[31,37] 또는 삼중 맹검을 수행하는 연구[36]가 있었는데, 이는 동일한 병 형태로 아로마 오일을 적용하거나 아로마 오일과 유사한 오일을 사용하는 플라시보 그룹을 사용했기에 가능하였다.

#### RoBANS 질 평가 결과

**Figure 3.**
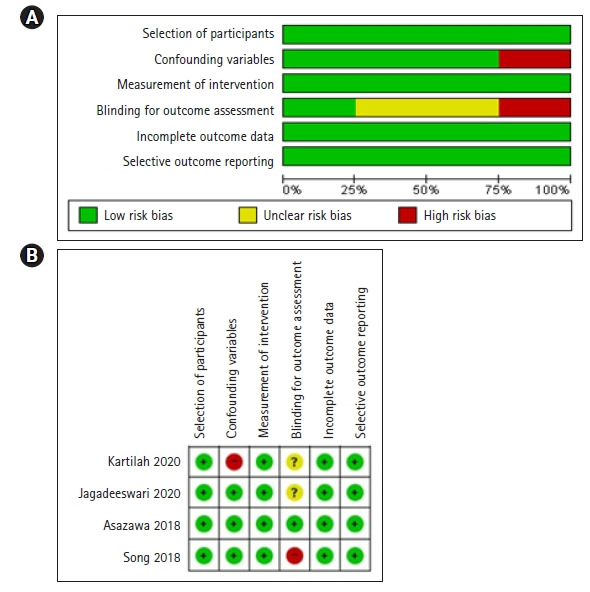
RIsk of bias for selected non-randomized studies. (A) Risk of bias graph and (B) risk of bias summary.

CCT 연구 4편의 문헌에 대한 질 평가는 다음과 같다(Figure 3). 대상군 선정과 중재 측정, 불완전한 자료, 선택적 결과 보고 항목에서 비뚤림 위험은 나타나지 않았으며, 교란변수 항목에서 낮은 위험을 가진 연구는 3편(75.0%) [21,38,39], 결과 평가에 대한 눈가림은 1편(25.5%) [38]으로 나타났다.

### 아로마테라피가 산후 여성의 피로에 미치는 영향

**Figure 4.**
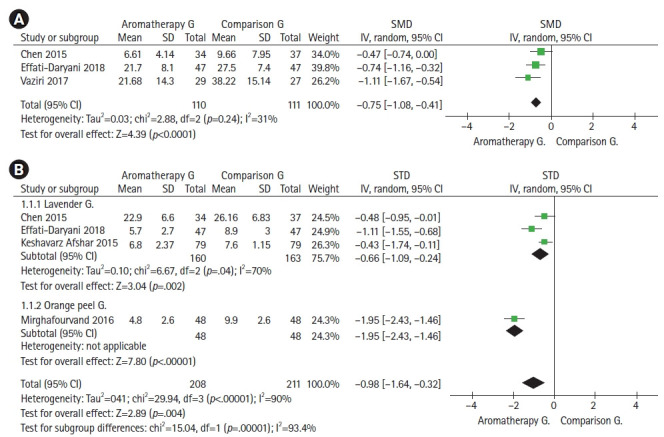
Forest plot of the effects of aromatherapy for fatigue and quality of sleep in postnatal women. The outcomes of (A) fatigue and (B) quality of sleep. df: Degree of freedom; SD: standard deviation; SMD: standardized mean difference; 95% CI: 95% confidence interval.

총 10편의 분석 논문 중에서 메타 분석이 가능한 3편[31,35,37]의 총 221명을 대상으로 아로마테라피가 산후 여성의 피로에 미치는 효과 크기를 분석하였다(Figure 4A). 아로마테라피를 적용한 군과 비교군을 분석한 결과 피로는 0.75점(SMD, –0.75; 95% CI, –1.08 to –0.41)의 감소효과가 있었고, 실험군과 비교군 간의 효과 크기는 통계적으로 유의한 차이를 보였으며(Z=4.39, *p*<.001) 이질성은 낮았다(Higgin’s I^2^=31%).

### 아로마테라피가 산후 여성의 수면의 질에 미치는 영향

총 10편의 분석 논문 중에서 메타 분석이 가능한 4편[31,35,38,39]의 총 419명을 대상으로 아로마테라피가 산후 여성의 수면의 질에 미치는 효과 크기를 분석하였다(Figure 4B). 아로마테라피를 적용한 군과 비교군을 분석한 결과 수면의 질은 0.98점(SMD, –0.98; 95% CI, –1.64 to –0.32)의 증진 효과가 있었고, 실험군과 비교군 간의 효과 크기는 통계적으로 유의한 차이를 보였으며(Z=2.89, *p*<.01) 이질성은 높았다(Higgin’s I^2^=90%). 이질성의 원인을 파악하기 위해 소그룹 분석을 시행하였다. 분석에 포함된 4편의 연구는 사용한 아로마 오일에 차이를 보였는데 3편[31,35,38]은 라벤더 오일을 사용하였고, 1편의 연구[39]에서는 오렌지 껍질(orange peel) 오일을 사용하였다. 라벤더 오일을 사용한 연구 3편에서의 수면의 질은 0.66점(SMD, –0.66; 95% CI, –1.09 to –0.24)의 증진 효과가 있었고, 효과 크기는 통계적으로 유의한 차이를 보였으며(Z=3.04, *p*<.01) 이질성은 중간 정도였다(Higgin’s I^2^=70%).

### 아로마테라피가 임신 여성의 피로와 수면의 질에 미치는 영향

임신 여성을 대상으로 한 연구는 총 3편이었다[31,34,40]. 라벤더 크림을 사용하여 하루에 1회, 총 6주 동안 적용한 하지 마사지는 임부의 피로와 수면의 질에 긍정적인 효과가 있었다[31]. 라벤더 오일 흡입 또한 산전 피로를 감소시켰으나 일회성 중재 연구에서는 통계적으로 유의한 차이를 보이지 않았으며[34], 일주일 동안 총 4회 흡입을 적용한 연구에서는 통계적으로 유의하게 감소하였다[40].

## Discussion

본 연구는 산전 및 산후 여성의 피로에 대한 아로마테라피의 효과를 확인하는 총 10편의 연구를 확인하였다[29-31,34-40]. 발표 연도는 2013년부터 2020년까지로 최근 산모들의 피로에 대한 관심이 높아진 것으로 보이며, 대부분 산후 산모를 대상으로 한 연구였다. 연구 설계는 RCT 6편[30,31,34-37], CCT 4편[29,38-40]으로 고도로 통제된 환경에서 아로마테라피의 효과를 검증하기 위한 노력이 지속되고 있음을 보여주었다. 또한 흡입 방법은 피로를 완화시키기 위한 가장 흔한 방향 요법이었으며, 그 다음으로 마사지 방법이었다. 475명의 대상자를 포함한 5개의 연구[31,35,37-39]가 메타 분석에 포함되어 산후 피로에 대한 중재 효과를 검토하였는데, 주요 결과에 대한 논의는 다음과 같다.

3편의 RCT 연구[31,35,37]에 대한 메타 분석의 결과는 아로마테라피가 산후 피로 해소에 효과적인 치료였다는 것을 제시했다. 분석에 포함된 아로마테라피의 빈도는 하루에 1회씩 1일, 2주, 6주로 다양하였으며, 중재는 10분에서 20분 동안 지속되었다. 3편의 연구에서 아로마테라피는 마사지, 흡입 및 차를 포함한 다양한 방법을 사용하여 시행했으며 공통적으로 라벤더 오일을 단독으로 사용하였다. 아로마테라피는 다양한 생리적 과정과 질병의 증상을 감소시키는 것으로 알려진 방법이며[41], 다른 요법의 보완제로 쓰거나 대체 요법으로도 사용할 수 있다. 그중 라벤더는 연구에 가장 자주 사용되는 필수 오일로 camphor, terpinen-4-ol, linalool, linalyl aetate, betaocimene, 1,8-cineole 성분을 포함하고 있다[42]. 이 성분들은 중추신경계의 활성을 떨어뜨려 진정 효과를 가지며, 불안과 수면장애를 줄이고, 행복감을 향상시킬 수 있는 것으로 알려져 있다[42,43]. 이외에도 몇몇 연구들은 라벤더를 이용한 방향 요법이 항염증, 항우울제, 최면, 진정제, 근육 이완제, 항균 등의 효과를 가지고 있다는 것을 보여주었다[41,44]. 라벤더 티백을 따뜻하게 마시는 것과 관련하여, 라벤더 차를 마신 실험군은 차를 마시지 않은 대조군보다 산후 피로 점수가 유의하게 낮아졌으나, 2주 후 사후 관리에서는 유의한 차이가 발견되지 않았다[35]. 이는 아로마 차의 긍정적인 효과가 중재 후 오래 지속되지 않았음을 의미한다. 따라서, 편의성을 고려하여 차의 지속적 효과를 평가하기 위해 매일 차를 여러 번 마시는 것을 권장할 수 있겠다. 그러나 라벤더는 안전한 오일이라고 보고되지만, 특정 아로마 오일을 장기간 사용했을 때 신장과 간에 만성 독성이 발생할 가능성은 배제할 수 없다[45]. 따라서 본 연구 분석 결과는 라벤더의 경구 섭취 효과를 확인할 수 있었으나, 잠재적인 부작용 측면에서 중재의 안전성에 대한 후속 연구가 필요하겠다.

4편의 RCT 연구[31,35,38,39]에 대한 메타 분석의 결과 역시 아로마테라피가 산후 수면의 질 향상에 효과적이었다. 아로마 오일의 진정 효과를 감안할 때, 아로마테라피는 수면 장애에 대한 대체 치료법이 될 수 있겠다. 특히 라벤더 오일이 수면의 질에 미치는 영향은 여러 연구에서 조사되었는데, 라벤더 아로마 오일을 베개에 적용하여 향을 흡입한 노인들은 수면의 질이 향상되었으며[46], 라벤더 오일을 사용한 마사지는 유아들의 수면을 돕는 것으로 나타났다[47]. 또한 라벤더 오일의 향은 중년 여성들의 불면증을 개선시켰으며, 수면의 질 향상에 효과가 있다고 보고되었다[48]. 본 연구의 분석 논문 중 1편의 연구[39]에서는 오렌지 오일의 향을 흡입하는 방법으로 사용되었다. 흔히 스위트 오렌지라고 불리는 Rutaceae과의 일종인 오렌지 오일은 부교감 신경계의 활동을 12% 증가시키고, 교감신경계 활동을 16% 감소시키며[49], 불안 및 피로, 불면증에 효과적인 오일이다[42]. 이러한 효능들이 산후 산모의 수면의 질 향상에 기여하는 것으로 보인다.

본 연구는 산후 여성에게 아로마테라피를 적용한 연구들을 대상으로 체계적 문헌고찰과 메타 분석을 통하여 개별적으로 효과 여부를 보고한 연구들의 결과를 보다 통합적이고 과학적으로 그 효과를 검증했다는 특면에서 그 의미를 찾을 수 있다. 특히 라벤더 및 오렌지 오일을 사용한 아로마테라피 요법이 피로를 줄이는 데 효과적이며 수면의 질을 향상시킬 수 있다는 것을 검증하였다. 즉, 에센셜 오일의 향 흡입과 마사지, 차 마시기 등을 통해 대상자의 이완을 증가시키고 피로를 감소시킬 수 있다 하겠다. 그러나 아로마 오일은 단독으로 적용하는 것보다 2, 3가지 오일을 혼합하여 사용할 때 상승 작용을 통해 효능이 극대화되는 시너지 효과가 일어난다고 알려져 있다[50]. 본 연구에서는 아로마 오일을 혼합하여 사용한 RCT 연구가 없어 그 효과 정도를 비교할 수 없었기에 추후 연구를 통한 검증이 필요하겠다.

호르몬의 변화와 혈액량의 증가는 임신 중의 피로를 초래한다[4]. 이러한 피로감은 보통 임신 기간에 따라 증가한다고 알려져 있는데, 이는 체중 증가와 성장하는 태아의 요구를 충족시키기 위해 신체기관의 활동이 활발해지기 때문이다[4]. 이러한 피로는 수면의 시간과 질에 따라 상당한 연관성을 가지고 있으며, 밤에 자는 시간이 낮잠보다 피로를 감소시키는 데 효과적이므로 임부에게 야간 수면을 권장해야 함을 주장한다[51]. 이외에도 임부의 불안과 우울, 스트레스도 피로에 영향을 주는 것으로 보고하고 있다. 아로마테라피는 손쉽게 실시할 수 있고, 비용이 많이 들지 않으며, 임부에게 많은 이점을 제공하는 중재 방법이다[52]. 페퍼민트(peppermint)와 라벤더, 생강(ginger)는 입덧을 완화할 수 있으며, 라벤더와 장미(rose)는 임부의 불안과 스트레스를 완화하는 것으로 밝혀졌다[52]. 본 연구에서는 임신 여성의 피로 연구에 대해 메타 분석을 통해 연구결과를 합성할 수 없었으나, 라벤더 오일을 이용한 흡입 및 마사지 요법은 산전 피로와 수면의 질에 긍정적인 효과가 있었음을 확인할 수 있었다[31,34,40]. 따라서 라벤더와 오렌지 오일은 산전 및 산후 여성의 피로 감소와 수면의 질 향상을 위해 적용될 수 있겠다.

본 연구는 몇 가지 제약이 있다. 가장 중요한 한계는, 모든 연구가 아로마 오일을 사용했지만 사용한 오일의 종류, 적용 방법 및 시간들이 다양했는데, 이는 연구 결과를 적용하는 데 중요한 제한점이라 할 수 있다. 또한 몇몇 연구에서는 언급된 개입에 대한 정확한 세부 사항을 제시하지 않아 더 엄격하게 분석하기가 어려웠으며[30,34,35,39,40], 메타 분석에 포함된 RCT 연구의 수가 매우 제한적이어서 그 결과를 해석하는 데 제한적이었다. 따라서 산전 및 산후 여성에 대한 아로마테라피 효과의 근거를 높이려면 라벤더, 오렌지 오일을 이용한 반복 연구와 더 엄격한 연구방법, 즉 RCT 방법을 적용하여 집중적인 효과 검증 연구가 필요하겠다. 이러한 제한점에도 불구하고 본 연구는 라벤더 및 오렌지 오일을 사용한 아로마테라피 요법이 산전 및 산후 여성의 피로를 감소시키고 수면의 질을 향상시킬 수 있다는 점을 확인하였다는 것에 의의가 있다.

## Figures and Tables

**Table 1.** Summary of randomized controlled trials examining the effects of aromatherapy on prenatal and postnatal fatigue (n=6)

First author (year) [reference]	Participants	Intervention group (regimen)	Control group (regimen)	Aromatherapy	Main outcome measures	Intergroup difference	Author’s conclusion
Effati-Daryani (2018) [31]	137 Prenatal and postnatal women	(A) Aroma cream leg massage 10–20 min, 1 time/day, pre-/postnatal 6 wk (n=47)	(C) Placebo cream leg massage 10–20 min, 1 time/day, pre-/postnatal 6 wk (n=44)	Lavender	(1) Fatigue: MAF	(1)-1. *p*<.001	“Lavender cream may improve sleep quality in pregnancy and postpartum.”
		(B) Aroma cream leg massage 10–20 min+footbath 30 min, 1 time/day, pre-/postnatal 6 wk (n=46)			(2) Sleep: PSQI	(1)-2. *p*<.001	
						(2)-1. *p*<.05	
						(2)-2. *p*<.001	
Vaziri (2017) [37]	56 Postnatal women	(A) Aroma oil inhalation 10–15 min (n=29)	(B) Placebo oil inhalation 10–15 min (n=27)	Lavender	(1) Fatigue: VAS	(1) *p*<.05	“Aromatherapy starting in the first hours of postpartum period resulted in better physical and mood status...”
					(2) Pain: VAS	(2) *p*<.001	
					(3) Mood: PANAS	(3) *p*<.05	
Mirghafourvand (2016) [36]	96 Postnatal women	(A) Aroma oil inhalation after meals, 3 times/day, 8 wk (n=48)	(B) Placebo oil inhalation after meals, 3 times/day, 8 wk (n=48)	Orange peel	(1) Sleep: PSQI	(1) *p*=.001	“Orange peel essential oil has a positive effect in improving mothers’ sleep quality in the postpartum period.”
							
Chen (2015) [35]	71 Postnatal women	(A) Aroma tea inhalation and drinking, 1 time/day, 2 wk (n=34)	(B) Routine care (n=37)	Lavender	(1) Fatigue: PFS	(1) *p*<.05	“The findings in this study can gain greater attention among healthcare practitioners and encourage the correct and positive use of herbal therapy in postpartum health care.”
					(2) Sleep: PSQS	(2) NS	
					(3) Depression: EPDS	(3) *p*<.05	
					(4) Bonding: PBQ	(4) *p*<.05	
Keshavarz Afshar (2015) [30]	158 Postnatal women	(A) Aroma oil inhalation during sleep, 4 days/wk, 8 wk (n=79)	(B) Placebo oil inhalation during sleep, 4 days/wk, 8 wk (n=79)	Lavender	(1) Sleep: PSQI	(1) *p*<.001	“…considering the effects of aromatherapy on the improvement of mother’s sleep quality during postpartum period...”
Igarashi (2013) [34]	13 Prenatal (28 wk) women	(A) Aroma oil inhalation 5 min (n=7)	(B) Routine care (n=6)	Lavender	(1) Mood: POMS	(1)-1. NS	“Aromatherapy inhalation using essential oils containing linalyl acetate and linalool was found to be effective for the POMS...”
					(1)-1. Fatigue	(1)-2. *p*<.05	
					(1)-2. Tension-Anxiety	(1)-3. NS	
					(1)-3. Depression	(1)-4. *p*<.05	
					(1)-4. Anger-Hostility	(1)-5. NS	
					(1)-5. Vigor	(1)-6. NS	
					(1)-6. Confusion		

EPDS: Edinburgh Postnatal Depression Scale; MAF: Multidimensional Assessment of Fatigue; NS, not significant; PANAS: Positive and Negative Affect Schedule; PBQ: Postpartum Bonding Questionnaire; PFS: Postpartum Fatigue Scale; POMS: Profile of Mood States; PSQI: Pittsburgh Sleep Quality Index; PSQS: Postpartum Sleep Quality Scale; VAS: visual analogue scale.

**Table 2.** Summary of controlled clinical trials examining aromatherapy on prenatal and postnatal fatigue (n=4)

First author (year) [reference]	Participants	Intervention group (regime)	Control group (regime)	Aromatherapy	Main outcome measures	Intergroup difference	Author’s conclusion
Kartilah (2020) [40]	52 Prenatal women	(A) Aroma oil inhalation+PMR, 4 days/wk	(B) PMR, 4 times/wk	Lavender	(1) Fatigue: VAS	(1) *p*<.05	“The results showed that aromatherapy with lavender oil was effective in reducing pain, fatigue, and pressure and could improve the mood of mother's.”
							
Jagadeeswari (2020) [39]	30 Postnatal women	(A) Aroma oil inhalation 15–20 min (n=15)	(B) Routine care (n=15)	Lavender	(1) Fatigue: FAS	(1) *p*<.001	“Aromatherapy as no side effects and it is easy and comfortable method which can be practiced to treat after pain and fatigue.”
					(2) Pain: VAS	(2) *p*<.001	
Asazawa (2018) [38]	229 Early postpartum (days 1–7) women	(A) Aroma oil hand massage 20 min (n=115)	(B) Routine care (n=114)	Participants selected one of lavender, ylang-ylang, citron, rosewood, sweet orange	(1) Fatigue	(1) NS	“The aroma hand treatment effectively promoted relaxation of early postpartum mothers, but was less effective in alleviating their fatigue.”
					(2) Relaxation	(2) *p*<.001	
Song (2018) [29]	32 Postnatal women	(A) Aroma oil hand massage, 2 times/day, 5 days (n=16)	(B) Placebo oil hand massage, 2 times/day, 5 days (n=16)	Lavender, bergamot, sandalwood	(1) Fatigue: Fatigue symptom checklist	(1) *p*=.001	“…aroma hand massage was effective in reducing the stress, fatigue, and depression of mothers in the postpartum period.”
					(2) Stress: PWI	(2) *p*<.05	
					(3) Depression: K-EPDS	(3) *p*<.05	

FAS: Fatigue Assessment Scale; K-EPDS: Koreanversion Edinburgh Postpartum Depression Scale; NS, not significant; PMR: progressive muscle relaxation; PWI-SF: Psychosocial Wellbeing Index-Short Form; VAS: visual analogue scale.
